# Cognitive vision system for control of dexterous prosthetic hands: Experimental evaluation

**DOI:** 10.1186/1743-0003-7-42

**Published:** 2010-08-23

**Authors:** Strahinja Došen, Christian Cipriani, Miloš Kostić, Marco Controzzi, Maria C Carrozza, Dejan B Popović

**Affiliations:** 1 Center for Sensory-Motor Interaction, Department for Health Science and Technology, Aalborg University, 9220, Aalborg, Denmark; 2 Advanced Robotics Technology and Systems Laboratory, Scuola Superiore Sant'Anna, 56025, Pontedera, Italy; 3 Faculty of Electrical Engineering, University of Belgrade, 11000, Belgrade, Serbia

## Abstract

**Background:**

Dexterous prosthetic hands that were developed recently, such as SmartHand and i-LIMB, are highly sophisticated; they have individually controllable fingers and the thumb that is able to abduct/adduct. This flexibility allows implementation of many different grasping strategies, but also requires new control algorithms that can exploit the many degrees of freedom available. The current study presents and tests the operation of a new control method for dexterous prosthetic hands.

**Methods:**

The central component of the proposed method is an autonomous controller comprising a vision system with rule-based reasoning mounted on a dexterous hand (CyberHand). The controller, termed cognitive vision system (CVS), mimics biological control and generates commands for prehension. The CVS was integrated into a hierarchical control structure: 1) the user triggers the system and controls the orientation of the hand; 2) a high-level controller automatically selects the grasp type and size; and 3) an embedded hand controller implements the selected grasp using closed-loop position/force control. The operation of the control system was tested in 13 healthy subjects who used Cyberhand, attached to the forearm, to grasp and transport 18 objects placed at two different distances.

**Results:**

The system correctly estimated grasp type and size (nine commands in total) in about 84% of the trials. In an additional 6% of the trials, the grasp type and/or size were different from the optimal ones, but they were still good enough for the grasp to be successful. If the control task was simplified by decreasing the number of possible commands, the classification accuracy increased (e.g., 93% for guessing the grasp type only).

**Conclusions:**

The original outcome of this research is a novel controller empowered by vision and reasoning and capable of high-level analysis (i.e., determining object properties) and autonomous decision making (i.e., selecting the grasp type and size). The automatic control eases the burden from the user and, as a result, the user can concentrate on what he/she does, not on how he/she should do it. The tests showed that the performance of the controller was satisfactory and that the users were able to operate the system with minimal prior training.

## Background

Most commercially available hand prostheses are simple one degree-of-freedom grippers [1,2] in which one motor drives the index and middle fingers synchronously with the thumb. The remaining fingers serve aesthetic purposes and move passively with the three active fingers. Recently, several dexterous prosthetic hand prototypes have been developed (e.g., SmartHand [3,4], HIT/DLR Prosthetic Hand [5], and FluidHand III [6]). Some hands are even commercially available (e.g., i-LIMB [7] and RSL Steeper Bebionic Hand [8]) or projected to appear on the market in the recent future (e.g., Otto Bock Michelangelo Hand [9]). In general, these are quite sophisticated devices that are morphologically and functionally closer to their natural counterpart. They have similar sizes and masses as the adult human hand, individually powered and controlled fingers, and a thumb that is able to abduct/adduct. The new devices ensure flexibility that allows implementation of many different grasps; yet, they require novel control algorithms that can exploit the many degrees of freedom available.

The control of an externally powered hand prosthesis is often implemented in the following manner [10,11]: 1) the user communicates his/her intentions (e.g., open or close the hand) by generating command signals; and 2) these signals are transferred to the hand controller, which decodes the signals, extracts the underlying commands, and drives the system. Following this general structure, the efforts to improve the control of hand prostheses have been directed towards increasing the bandwidth of the communication link between the user and the system, i.e., increasing the number of commands that can be generated by the user and recognized by the controller.

Different types of signals (e.g., electromyography (EMG) [12], voice [13], insole pressures [14], muscle and tendon forces [15]), and pattern recognition signal processing techniques (e.g., artificial neural networks, fuzzy and neuro-fuzzy systems, Gaussian mixture models, linear discriminant analysis, and hidden Markov models [12,16-24]) have been suggested and tested for this purpose. A characteristic of these methods is that the result depends on the ability of the user to generate distinct commands in a reproducible manner. The user needs to go through a training program in order to learn how to use the system. As a rule, the more sophisticated the system is, the more conscious the effort and attention that is needed to operate it, especially if the control interface is less intuitive (e.g., voice [13], insole pressures [14]). Finally, as Cipriani *et al. *[25] showed, although more sophisticated control allows better performance, the preference of the user is to use the simple, less effective control, since it does not require conscious involvement ("how to use the device"). This is one of the major reasons why most of the commercially available prosthetic hands (e.g., Otto Bock Sensor Hand, Touch Bionics i-LIMB, and RSL Steeper Bebionic) implement simple myoelectric control: a surface EMG is recorded from at most two sites on the residual limb and used as a proportional or discrete (ON/OFF) input for the control of opening and closing of the hand [26,27].

The main challenge is therefore how to implement more sophisticated control (e.g., many commands and/or independently controlled degrees of freedom) without simultaneously overburdening the user. This could be achieved by means of recently introduced promising surgical procedures and techniques, such as the Targeted Muscle Reinnervation proposed by Kuiken *et al. *[28,29].

A non-invasive approach for decreasing the burden to the user is to make the artificial hand controller more autonomous. This idea has been proposed originally by Tomović *et al. *[30,31] in 60's and implemented within the Belgrade Hand. The hand was instrumented with pressure sensors, which were used for the semi-automatic selection of the grasp type based on the point of initial contact with the object. If the initial contact was detected at the fingertip, the pinch grasp was triggered. Otherwise, if the contact was at the palm or along the first phalanx, the palmar grasp was executed. Nightingale *et al. *[32-35] improved and extended this concept by implementing it within a hierarchical control scheme. The user issued high level commands (open, close, hold, squeeze and release), and the controller was capable of selecting precision or power grasp (touch sensors), performing the selected grasp, and holding an object with the minimal required force (slippage sensors).

In this manuscript we propose an autonomous controller that is empowered by artificial vision and reasoning. The reasoning that we advocate is borrowed from the human motor control [36-38]. The sensorimotor systems of a human, when grasping, builds the opposition space and orients the hand to match the opposition space of the hand to the object. This yields to the posture (grasp type) in which a set of balanced forces is applied to the object surfaces, resulting in force equilibrium. In humans, the reasoning of how to orient the hand and build the opposition space is developed through learning and critically depends on the vision [37].

Beginning with the work of Cutkosky, researches have demonstrated that it is possible to predict the type of grasp from the object properties and task requirements by employing a set of rules [39] or artificial neural networks [40]. Tomović *et al. *[41] suggested using rules to select a grasp type for an artificial hand prosthesis based on the estimated object size. Iberall *et al. *[42] designed the control for a simulated artificial hand in which a myoelectric interface was used to choose from the three hand postures (pad, palm, and side opposition), each one available in several predefined aperture sizes.

The authors have recently developed a cognitive vision system (CVS) that uses computer vision and rule-based reasoning to automatically generate preshaping and orientation commands for the control of an artificial hand [43]. The CVS employs a standard web camera and a distance sensor for retrieving the image of the target object and measuring the distance to it. This information is used to estimate the size and orientation of the object, and these estimates are then processed by employing heuristics expressed in the form of rules in order to select an appropriate grasp type, aperture size and orientation angle for the hand (for details see [43]).

In this paper, we demonstrate how the CVS can be integrated into a hierarchical control structure for the control of a dexterous prosthetic hand. The operation of the system was tested in 13 healthy subjects. The CyberHand prototype [44] was mounted onto an orthopaedic splint and attached to the forearm of each subject, thereby emulating the use of a prosthetic hand. The goal of the current study was to test the feasibility of the proposed control method, in particular the feasibility of integration of the autonomous artificial control with the volitional (biological) control of the user. This is an essential step before evaluating the usability of the suggested approach for the control of a functional transradial prosthesis operated by an amputee. The results in this paper refer to the efficacy of grasping the objects, typical for daily activities, placed at different positions within the workspace.

## Methods

### Control system architecture

**Figure 1:**
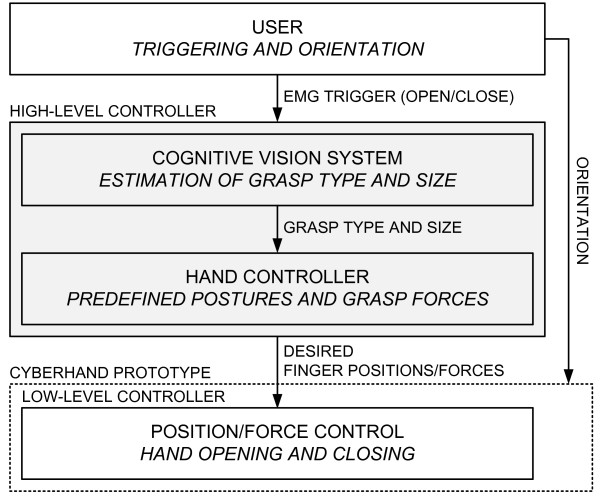
**Control system architecture**. The Cognitive Vision System (CVS) is integrated into a hierarchical control system for the control of a dexterous prosthetic hand (emulated by the CyberHand prototype). The user triggers the system and controls the orientation of the hand. A high-level controller autonomously selects the grasp type and size that are appropriate for the target object. A low-level controller embedded into the hand provides a stable interface for preshaping and grasping.

The conceptual scheme of the implemented control is depicted in Fig. 1. It is a hierarchical structure, in which the overall control task is shared between the user, a high-level controller and a low-level embedded controller. The user issues commands for hand opening and closing via a simple EMG interface and also controls the orientation of the hand during grasping and manipulation. The high-level controller comprises: 1) the CVS estimating object properties (size, shape) and automatically selecting grasp type and aperture size appropriate for grasping the object; and 2) a hand controller translating the selected grasp into a set of desired finger positions (for hand preshaping) and forces (for hand grasping) that are sent to a low-level controller. The low-level controller embedded into the CyberHand prototype implements closed-loop position and force control during hand preshaping and grasping, respectively. The novel contribution of this study is the development of the high-level controller and the integration of the aforementioned elements into a unified control framework.

### Experimental setup

**Figure 2:**
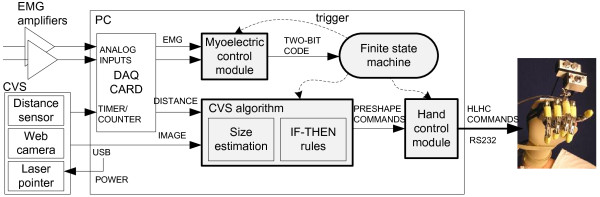
**The implementation of the control system architecture**. The hardware comprises: 1) the cognitive vision system (CVS), 2) a two-channel EMG system, and 3) a PC with a data acquisition card. The PC runs a control application implementing a finite state machine that triggers the following modules (gray boxes): the myoelectric control module, the CVS algorithm and the hand control module. The myoelectric module acquires and processes the EMG, generating a two-bit code signalling the activity of the flexor and extensor muscles. This code is the input for the state machine. The CVS algorithm estimates the size of the target object and uses a set of simple IF-THEN rules to select the grasp type and aperture size appropriate to grasp the object. The hand control module implements the selected grasp parameters by sending the commands to the embedded hand controller (HLHC) via an RS232 link.

**Figure 3:**
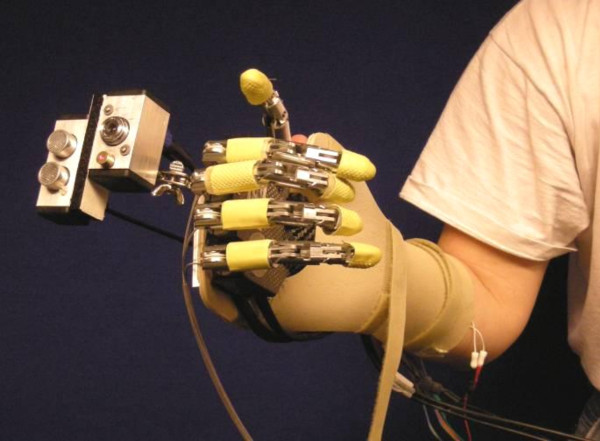
**Experimental platform**. The platform consists of: 1) the CyberHand attached onto an orthopaedic splint, 2) the cognitive vision system (CVS) mounted onto the dorsal side of the hand via a pivot joint, and 3) the EMG electrodes for myoelectric control.

The experimental setup consisted of the following components (see Fig. 2): 1) the prosthetic hand mounted onto an orthopaedic splint, 2) the CVS, 3) a two-channel EMG system, and 4) a standard PC (dual-core Pentium 2 GHz) equipped with a DAQ card (NI-DAQ 6062E, National Instruments, USA). The control was run within an application developed in LabView 2009. As can be seen from Figs. 2 and 3, the hand was rigidly fixed for the orthopaedic splint (no wrist joint) and the splint was attached to the subject's forearm by using straps, in such a way that the artificial hand was just below the subject's hand and oriented in the same manner (i.e., the palm of the artificial hand was parallel to the volar side of the subject's forearm). The subject could rotate the artificial hand by using pronation/supination.

#### Prosthetic hand

The stand-alone version of the CyberHand prototype [44], already employed in many research scenarios [25,45,46], was used to emulate a prosthetic hand. It consists of four under-actuated anthropomorphic fingers and a thumb based on Hirose's soft finger mechanism [47] and actuated by six DC motors. Five of them, located remotely, control finger flexion/extension. One motor, housed inside the palm, drives the thumb abduction/adduction. The hand is comparable in size to the adult human hand, and the remote actuators are assembled in an experimental platform that mimics the shape of the human forearm. The remote actuators act on their respective fingers using tendons and a Bowden cable transmission. Active flexion is achieved as follows: when a tendon is pulled, the phalanxes flex synchronously, replicating the idle motion (i.e., free space motion) of a human finger [48]. As a result of this mechanism, the shape of the hand adapts to the shape of an object automatically, providing multiple contact points and a stable grasp. Therefore, the final geometrical configuration of the hand is dictated by external constraints imposed by the shape of the grasped object. When a tendon is released, torsion springs located within the joints extend the fingers, thereby providing hand opening and releasing of the object.

The hand includes encoders integrated in the motor units (position sensors) and force sensors in series with the tendons (for the assessment of the grasp force).

The controller embedded in the hand (low-level controller in Fig. 1) is an 8-bit, microcontroller-based architecture (Microchip Inc. microcontrollers); it is itself organized in a hierarchical manner and consists of six low-level motion controllers (LLMCs) and one high-level hand controller (HLHC). Each motor is directly actuated and controlled by an LLMC that implements a proportional-integral-derivative (PID) position control and force control based on tendon tension. All LLMCs are directly controlled by the HLHC, which regulates overall hand operation and acts as an interface with the external world. This interface comprises a set of commands that can be sent to the hand from a host PC via a standard RS232 serial link. It includes commands for reading the forces and positions, as well as for setting the finger positions in the range from 0 (fully open) to 100% (fully flexed) and tendon forces in the range from 0 (no force) to 100% (maximal force ~140 N).

#### Cognitive vision system (CVS)

The CVS is composed of a small-sized, low-cost web camera (EXOO-M053, Science & Technology Development Co. Ltd., China), an ultrasound distance sensor (SRF04, Devantech Ltd., UK) and a laser pointer, housed in a custom-made metal housing, mounted onto the dorsal side of the hand using a pivot joint (see Fig. 3) and communicating with a PC via a DAQ card and USB port [43]. Two timer/counter modules on the DAQ card were used to interface with the distance sensor: one to generate a trigger pulse to start the measurement and the other to read the pulse-width-modulated (PWM) sensor output. The web camera was connected directly to a USB port of the PC, whereas the laser pointer was simply powered by using the power lines of the USB interface. The laser pointer was used to point at the object that was the target for grasping, the web camera provided the image of the object and the distance sensor measured the distance to the target.

#### EMG system

Bipolar EMG was recorded from the finger flexor (flexor digitorum superficialis and profundus) and extensor muscles (extensor digitorum communis) by using standard, disposable, self-adhesive Ag/AgCl electrodes (size 3 × 2 cm, Neuroline 720, AMBU, SE). The outputs of the EMG amplifiers were connected to the analog input channels of the DAQ card. Single-channel isolated EMG amplifiers (EM002-01, Center for Sensory-Motor Interaction, DK) were used. The input channel (CMRR >100 dB, input impedance >100 MΩ, gain ≤10000) was equipped with an analogue second-order band-pass Butterworth filter with the cut-off frequencies set at 5 and 500 Hz. The amplifiers were custom made at the Centre for Sensory-Motor Interaction and used previously in a number of motor control studies.

#### Control algorithm

The control algorithm integrates the following tasks: 1) acquires input information: image and distance from the CVS, and EMG signals from the amplifiers, 2) processes the data, 3) generates hand control commands, and 4) sends them to the hand. The control application implements a finite state machine in which transitions between the main states (hand open and close) are triggered by the user's EMG. The processing part, i.e., the core of the application, comprises three distinct modules: the CVS algorithm, the myoelectric control and the hand control modules (see Fig. 2).

The CVS algorithm processes the image and distance information. In the first stage, computer vision methods [43] are used to analyze the image in order to locate the target object and to estimate its size, i.e., the lengths of its short and long axes. The size is estimated using the distance to the object (as measured by the distance sensor), the length of the object axes in pixels, and the focal length of the camera [43]. When the user triggers the operation of the CVS (as explained later), ten consecutive measurements are performed. The final size estimate is obtained as the median of these ten estimates. The median is used in order to obtain more robust estimation, since it is less affected by potential outliers compared to the mean value.

**Figure 4:**
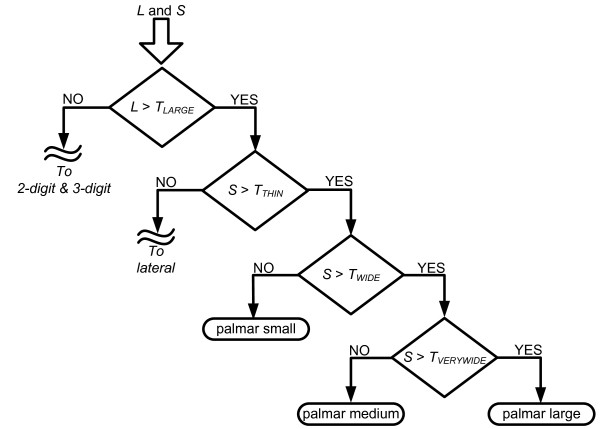
**A decision tree depicting the IF-THEN rules for the selection of the grasp type and size**. The inputs for the rules are the estimated lengths of the object's short (*S*) and long (*L*) axes. The lengths are compared against fixed thresholds (*T*) by following decision nodes (diamond shapes) of the tree until one of the leaf nodes (rounded rectangles) is reached. The thresholds are defined relative to the hand size and the size of the maximal aperture when the hand is preshaped according to a given grasp type. For example, *T*_*LARGE *_= 90% *PW*, *T*_*THIN *_= 70% *MLA*, *T*_*WIDE *_= 50% *MPA*, and *T*_*VERYWIDE *_= 65% *MPA*, where *PW *is the width of the palm (from lateral to medial side), while *MPA *and *MLA *are the maximal aperture sizes for the palmar and lateral grasps, respectively. For the full set of rules see the additional file 1.

The estimated object size is input for the cognitive part of the algorithm that is implemented as a set of IF-THEN rules. These rules compare the estimated size against fixed thresholds (IF) and based on the results of the comparisons, an appropriate grasp type and aperture size is selected (THEN). The rules are constructed so that four different grasp types can be chosen: *palmar*, *lateral*, *3-digit *and *2-digit *(pinch) grasps. Furthermore, *palmar *and *lateral *grasps are available in three different aperture sizes (*small*, *medium*, and *large*) while the *3-digit *grasp has two available sizes (*small *and *medium*). Therefore, there are nine possible grasp modalities in total (see Table 1). The main principle in designing the rules was to match the size of an object with a corresponding functional grasp; large objects trigger the selection of *palmar *or *lateral *grasps, whereas the *3-digit *and *2-digit *grasps are used for small and very small objects, respectively. If a large object is also wide enough, a *palmar *grasp is chosen; otherwise, for thin objects, a *lateral *grasp is used. The qualitative terms of "small", "large", "wide" and "thin" are quantified using numerical thresholds, and the thresholds are expressed in the percents of the hand size and the size of the maximal aperture when the artificial hand is preshaped according to a given grasp type. As an example, Fig. 4 shows the rules used for the palmar grasp. Rules for the other grasps are very similar (see the additional file 1). Importantly, different grasps are mutually exclusive, i.e., only one output can be generated by the CVS algorithm for the given input.

**Table 1:** Grasp types and sizes

Type of opposition	Grasp type and aperture size	Grasp ID
Palm opposition		
All palmar surfaces of the fingers and the palm are involved and the thumb is in opposition to other fingers (as in grasping a bottle).	Palmar Large	PL
	Palmar Medium	PM
	Palmar Small	PS

Side opposition		
The thumb opposes the radial aspect of the index finger (as in grasping a key).	Lateral Large	LL
	Lateral Medium	LM
	Lateral Small	LS

Pad opposition		
The opposition is formed between the fingertips of the thumb and the fingers (as in lifting a pin from a flat surface).	3-digit Medium (index, middle finger and thumb)	TM
	3-digit Small	TS
	2-digit (index finger and thumb)	B

**Figure 5:**
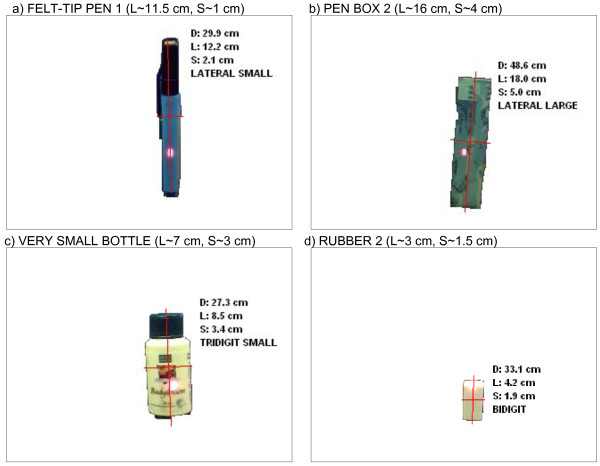
**The representative outputs of the cognitive vision algorithm**. The images depict the detected target object (see Table 2), measured distance (*D*), estimated lengths of its short (*S*) and long (*L*) axes and estimated grasp type and aperture size. The actual object sizes are given above the images. The estimated object axes are also shown graphically (superimposed gray lines). The bright spot is the reflection of the laser beam. The figure demonstrates that the cognitive vision system estimates the grasp types and sizes that are appropriate for the size of the target object. (Notations: Bidigit ~2-digit grasp, Tridigit ~3-digit grasp)

To demonstrate the operation of the CVS, we show in Fig. 5 the representative outputs of the CVS algorithm obtained during the experiments described later in the text. Pictures shown in Fig. 5(a)-(d) were generated when the CVS aimed at different target objects used in this study. Each image shows the detected object, the measured distance (*D*), the estimated lengths of the short (*S*) and long (*L*) object axes, and the resulting grasp type and size selected. For example, the object in Fig. 5(a) is long and thin, and the estimated grasp type was therefore *lateral*. The CVS selected the same grasp type for the object in Fig. 5(b), but since this time the object was wider, the estimated aperture size was *large*. Fig. 5(c) shows a small object for which the selected grasp was *3-digit small *and for the smallest object in Fig. 5(d), the estimation was *2-digit *grasp.

The prehension control commands generated by the CVS algorithm are inputs for the hand control module. The task of this module is to send the proper HLHC commands to the hand in order to preshape or close the hand according to the output of the CVS. A lookup table with the preshaping positions and tendon force values (for stable grasps) that should be assumed by each finger in each grasp was built. Values were chosen based on Cutkosky's grasp taxonomy [39], i.e., the forces were set according to the expected power demands in different grasps (e.g., higher forces for *palmar *than for *2-digit *grasp, higher forces for larger aperture sizes, etc.).

**Figure 6:**
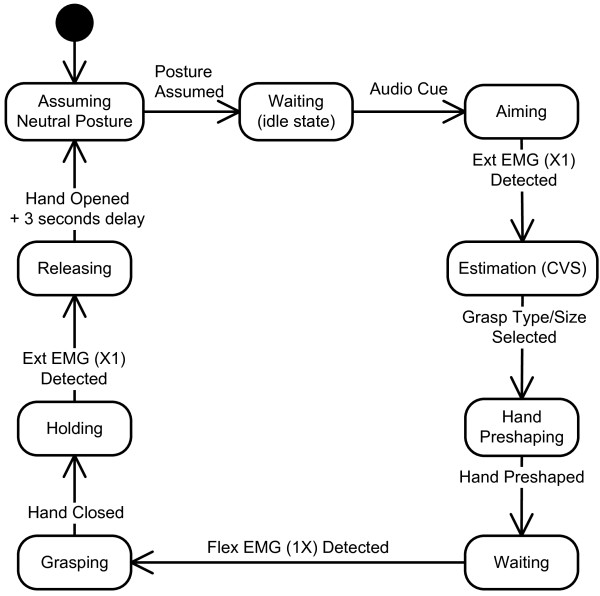
**Finite state machine for the control of the artificial hand**. The control is realized as an integration of the cognitive vision system (CVS) with myoelectric control. The two channels of electromyography (EMG) recorded from finger extensors (Ext EMG) and flexors (Flex EMG) drive the system through the states by providing a two-bit binary code (in brackets); the first bit signals the activity of the flexors and the second is for the extensors, while X means "don't care." The user aims the system toward a target object and triggers the hand opening. The CVS estimates the grasp type and size. The user reaches for the object, commands the hand to close, manipulates the object and finally commands the hand to open and release the object. Notations: rounded rectangles - states; full black circle - entry state; arrows - state transitions with events.

The myoelectric control module simply thresholds the EMG inputs in the following manner: raw EMG signals are sampled at 2 kHz, and the mean absolute value (MAV) is calculated over 100-ms overlapping time windows. The MAVs of both channels are then thresholded using individually adjustable levels, and a two-bit binary code (first bit referring to flexor muscles and second to extensors) is generated. The binary code is input for the application's state machine (see Fig. 6) implementing the following steps:

1) The starting, idle state is where the robotic hand is in a neutral posture (i.e., all fingers 60% flexed).

2) When the subject decides to grasp an object, he/she needs to point with the laser beam toward the object and activate his/her finger extensor muscles. The recognized EMG activity that is larger than the preset threshold starts the CVS algorithm for the estimation of the pointed object size and selection of the appropriate grasp type and aperture size.

3) Once the size and grasp type are selected, the hand control module commands finger extension, thereby providing preshaping.

4) The subject then grasps the object by positioning the hand around the object and commanding its closure by activating his/her finger flexors. The artificial hand grasps the object by using force control to flex the involved fingers.

5) The object is held until the subject contracts his/her finger extensor muscles, thereby triggering the opening of the hand and releasing of the object.

6) The final phase is the return to the idle state (after a three-second delay).

### Experimental protocol: "reach, pick up and place" trials

**Figure 7:**
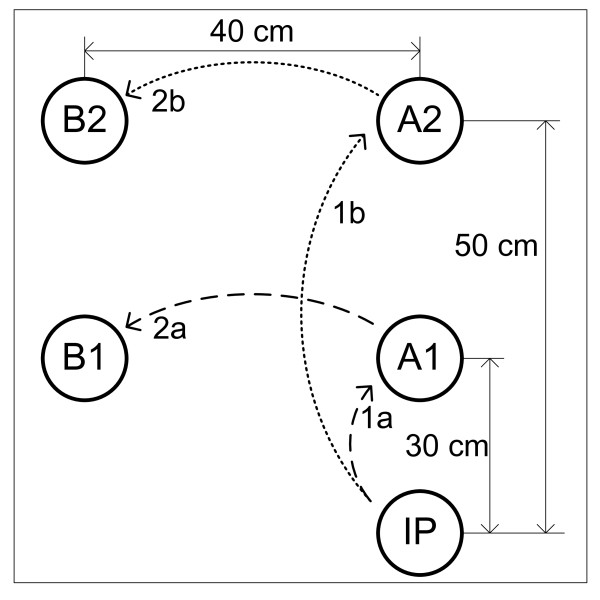
**Experimental workspace**. The notations are: IP - initial position for the hand; A1, A2 - initial positions for the object to be grasped; B1, B2 - target locations for the object placed at A1 and A2, respectively. The task for the subject was to reach for an object, grasp it, transport it to the target location and release it. Two sequences were used depending on the initial position of the object: IP-A1-B1 and IP-A2-B2.

The working principle of the system was tested in experimental trials in which subjects operated the artificial hand in the "reach, pick up and place" tasks. 13 able-bodied subjects participated in the experiments (29 ± 4.5 years of age). All volunteer subjects signed the informed consent approved by the local ethics committee. The subjects were comfortably seated on an adjustable chair in front of a desk where a workspace was organized (see Fig. 7). The workspace comprised a plane background with five positions marked: the initial (rest) position for the hand (labelled IP), two positions (A1 and A2) where the objects to be picked up were placed, and two positions (B1 and B2) to which the objects had to be transported; B1 and B2 were used as the final positions if the object was initially at A1 or A2, respectively. The positions A1 and A2 were 30 cm and 50 cm away from the initial position, respectively.

18 objects listed in Table 2 were selected as targets; the objects were chosen in order to have two samples for each of the grasp types given in Table 1. The task was to reach, grasp, transport and release the target object by operating the artificial hand as explained in the previous section. The subject was instructed to place the hand on the initial position so that the ulnar side of the hand rested on the table. Upon receiving an auditory cue, he/she had to drive the system through all of the states of the state machine by using myoelectric control, as shown in Fig. 6. During aiming, the subject was told to orient the hand so that the palm was facing down, parallel to the surface of the table. This orientation was selected to ensure that the CVS operated in identical conditions during the experiment, and also because during the preliminary tests, the subjects reported that this orientation was the easiest for aiming. After the CVS finished processing and the hand started preshaping, the subjects were free to move the system in any way desired. There were two blocks of 18 trials for each subject. In the first block, the target objects were placed at the location A1 (i.e., the sequence was IP-A1-B1), while in the second block, the location was A2 (i.e., the sequence was therefore IP-A2-B2). In both blocks, the target objects were selected in a random order. In order to minimize muscle fatiguing due to the perceived weight of the prosthesis (about 300 grams for the prosthesis and about 100 grams for the CVS on a longer lever-arm, compared to the natural hand), there was a five-minute resting period between the two blocks.

**Table 2:** Target objects

Grasp ID	Object	Size of the back plane projection *S *× *L *[cm]	Mass [g]
PL	Cylinder	10 × 18	650

PL	Cylinder	11 × 17	600

PM	Big cup	8 × 9	280

PM	Big bottle	8 × 25	550

PS	Spray Can	6 × 12	220

PS	Small bottle	6 × 22	480

B	Rubber 1	1 × 1.5	10

B	Rubber 2	1.5 × 3	15

TS	Lego element	3 × 5.5	10

TS	Very small bottle	3 × 7	30

TM	Tennis Ball	6	60

TM	Light bulb box	5 × 5	70

LS	Felt-tip pen 1	1 × 11.5	20

LS	Pen	1 × 13	25

LM	Felt-tip pen 2	2.5 × 11.5	30

LM	Pen box 1	2.5 × 16	40

LL	Pen box 2	4 × 16	35

LL	Plastic box	3.5 × 13	80

Notations: *S*, *L *- short and long axes, respectively.

Two of the subjects participated in a longer experiment comprising four extra blocks (six in total, alternating between A1 and A2) of 18 trials separated by five-minute breaks in order to better analyze improvements in performance due to learning.

At the beginning of the experiment, the amplifier gains and EMG thresholds were set to meet individual abilities of each subject. The subjects practiced the use of the system for about ten minutes. Attention during practicing was primarily paid to the proper pointing of the laser beam towards the object and to generating the appropriate muscle contractions of the finger extensors and flexors above the preset thresholds.

The following outcome measures have been used to evaluate the performance: 1) estimation accuracy: the estimation was considered successful if the grasp type and size were estimated according to the classification given in Table 2; 2) task accomplishment: the task was considered accomplished if the object was correctly picked up, transported and placed at the target location (as in [25]); and 3) the total time spent to accomplish the task. In the analysis, we considered that the task accomplishment and successful estimation are not directly related. Namely, the task could be accomplished even though a wrong grasp was used (e.g., lateral grasp to pick up a bottle); on the other hand, the subject could fail to do the task despite the fact that the grasp was successfully estimated (e.g., the object slipped).

Statistical differences among experimental results were evaluated using the Wilcoxon signed rank test for comparing two groups with paired data (i.e., repeated measurements) and the Friedman test for the simultaneous comparison of more than two groups with paired data. If the Friedman test suggested that there was a difference, groups were compared pairwise using the Bonferroni adjustment. Non-parametric tests were used since the collected data did not pass the tests for normality (e.g., Lilliefors test). Due to the same reason, median and inter-quartile ranges were selected as the summary statistics for the data. The groups for the statistical analysis were formed based on the blocks of trials. For example, the results achieved in the first block (group 1) were compared with the results obtained in the second block (group 2). The data from two different groups were paired based on the same target object and/or subject. For example, the time spent to grasp and transport a small cup in the first block (a result from group 1) was paired with the time spent to grasp and transport the same object in the second block (a result from group 2). A level of *p *< 0.05 was selected as the threshold for the statistical significance. The statistical analysis was performed using MatLab 2009b (The MathWorks, Natick, MA, USA) scripts.

## Results

13 subjects performed a total of 612 grasp trials; among these, 11 subjects performed 2 blocks of 18 trials, and 2 subjects performed 6 blocks of 18 trials. Overall, the CVS correctly estimated both grasp type and grasp size in 84% of the cases. In an additional 6% of the cases, the estimation was wrong but the task was still successfully accomplished. Two different errors were observed here. In half of the cases, the grasp type was correctly estimated but the grasp size was actually larger than the correct one. For example, the CVS estimated *palmar large *for an object that was supposed to be classified as a *palmar medium *grasp. Obviously, this type of error could not jeopardize the task accomplishment. In the other half of the cases, the estimated grasp type was actually wrong, but it was still similar enough to accomplish the task. For instance, instead of using the *2-digit *grasp for a very small object, the CVS estimated *3-digit small*. Therefore, from the functional point of view, the estimation was successful in about 90% of the trials.

**Figure 8:**
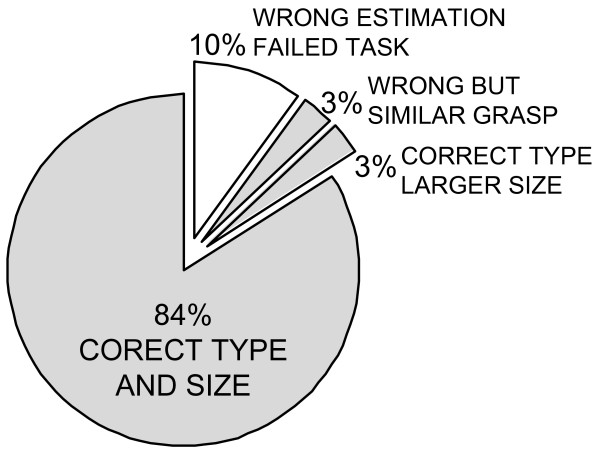
**Overall estimation accuracy for the grasp type and size**. Both grasp type and size were correctly estimated in 84% of the cases. In 3% of the cases, the type was correct and the size was larger than the correct one. We had the same number of cases (3%) in which the grasp was wrong but still similar enough for the subject to accomplish the task. Therefore, from the functional point of view, the classification was successful in 90% of the cases (all gray slices).

**Figure 9:**
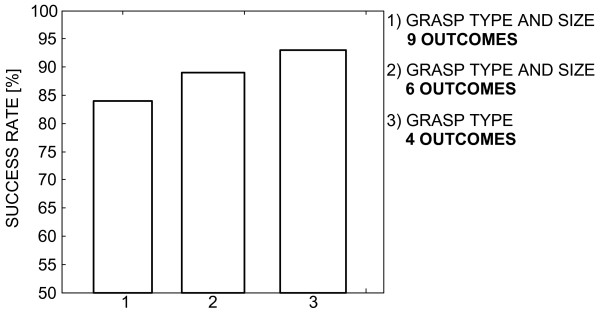
**Classification accuracy for different number of possible outputs**. If the number of possible outputs (i.e., hand preshape commands) that the IF-THEN rules can generate is decreased, the success rate improves. Groups: 1 - all grasp types and sizes, 2 - two grasp sizes for the lateral and palmar grasps and one grasp size for the 3-digit and 2-digit grasps; 3 - only grasp types (i.e., one grasp size for all grasp types).

No statistical difference between the estimation accuracies obtained for the two different distances (i.e., IP-A1 and IP-A2) was found. Importantly, if the number of choices in the rule-based classification was decreased, the success rate improved. For example, if the output was limited to just two sizes for the *lateral *and *palmar *grasps and a single size for the *3-digit *grasp (i.e., merging *medium *and *small *grasps), the classification was successful in 89% of the cases. Finally, if considering the grasp type only (regardless of the grasp size), the success rate was 93%. The results achieved in this study are summarized in Figs. 8 and 9.

From the point of view of successful task accomplishment, 5 out of 13 subjects showed an improvement between the second and first blocks of trials. The subject that showed the best improvement failed five times in the first block and just once in the second block of trials. Considering the whole group, the total number of unsuccessful tasks decreased from 27 in the first block to 20 in the second. Two subjects who performed six blocks had no failures in the last block of trials. For the above analysis, only the trials that were unsuccessful despite the fact that the grasp type and size were correctly estimated were taken into account (otherwise, the responsibility for the failure was attributed to the CVS).

The analysis on a subject by subject basis showed that in 10 out of 13 subjects, the median time spent to accomplish the task decreased in the second block of trials. Maximal registered improvement was 4.45 seconds. In eight of these ten subjects, the change was statistically significant. When regression lines were fitted through the data for each subject organized across the trials, the line slope was negative in 11 subjects, suggesting a trend for the decrease in time during the course of the experiment. When the first and second blocks were compared by considering the whole group (all subjects), the median decreased from 17 to 14.9 seconds, and this change was statistically significant.

**Figure 10:**
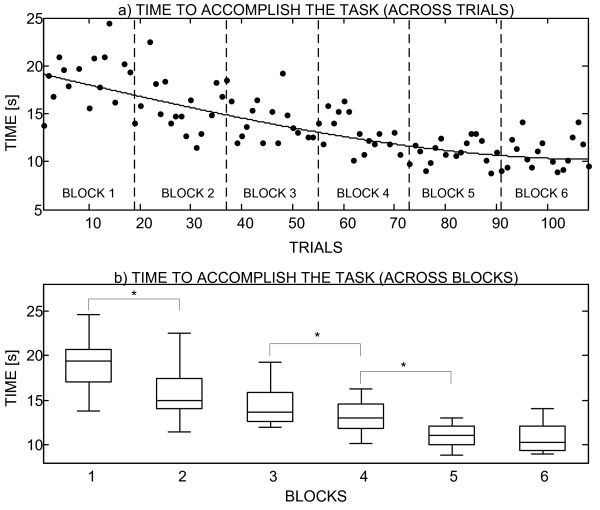
**Improvements in performance due to learning**. The figure shows the results (time spent to accomplish the task) organized as a) individual trials and b) blocks of trails. The vertical axis is the time needed to accomplish the task. In plot a), the trend obtained by fitting a cubic polynomial through the experimental results (black dots) is shown by a continuous line, and the boundaries between the blocks of trials are depicted by the dashed vertical lines. In plot b), the horizontal lines are the medians, boxes show inter-quartile ranges and whiskers are minimal and maximal values. Statistically significant difference is denoted by a star. The time needed to successfully accomplish the task decreases steadily during the experiment.

Fig. 10 clearly shows the improvement in performance throughout the experiment for one of the subjects that took part in the longer evaluation (i.e., 6 blocks × 18 trials); results for the second subject were comparable but for a better readability of the graph they are not included. The plot in Fig. 10(a) presents the time spent to accomplish the task versus the trial number. A cubic polynomial was fitted to the data to show the trend: time decreased and this decrease was slowing down. If the times are compared between the consecutive blocks, paired by the target object (Fig. 10[b]), then the median time in the first block was 19.4 seconds and it dropped to 10.3 seconds in the last block.

## Discussion

The goal of this study is to present and assess a novel concept for the control of grasping in transradial prostheses. The core of the presented architecture is the cognitive vision system (CVS) that uses artificial vision and a rule-based decision making to analyze the target object and to generate proper commands for the control of prehension. The tests showed that the autonomous artificial controller was successfully integrated with the biological control of able-bodied users. The CVS was combined with a simple EMG interface resulting in a fully functional prototype of an artificial hand operated by means of a shared (cooperative) control. The user was responsible for aiming, triggering, and orienting the hand while the automatic control implemented the selection of the grasp type and size, hand preshaping (position control) and grasping (force control). The prototype was successfully tested in healthy subjects that used it to grasp, transport and release a set of common objects. The current results (i.e., short training, success rates, and overall user impression) imply that the proposed concept might be successfully translated to the control of a dexterous prosthetic hand operated by amputees.

The controller designed in this study is capable of making high-level decisions autonomously. As a result, the communication link between the user and the system is very simple; the user issues just the basic commands (e.g., triggering grasp and release), and the controller implements the rest. Importantly, since the CVS is a self-contained component that uses a novel type of feedback (i.e., artificial vision and ultrasound), it can be combined/integrated with the other aforementioned control schemes described in the literature (e.g., [16,32,49,50]).

The use of the system is intuitive. The subject activates hand preshaping by contracting his/her finger extensors and then closes the hand by activating the finger flexors, which follows the healthy-like grasping muscle activation. The automatic control eases the burden from the user and, as a result, the user can concentrate on what he/she does and not on how he/she should do it. The other quality is that the intuitive control allowed the operation with virtually no prior training. In the present experiment, less than 15 minutes of practicing were enough for the subjects to start using the system successfully. During that time, the subjects learned to generate proper EMG signals, to aim at the target object using a laser beam, and to orient the hand for grasping and during manipulation. However, the current test was conducted in healthy subjects. In transradial amputees, who are the actual target population, setting up the EMG interface and learning its use could take more time depending on the state of the residual limb (i.e., severity of the damage). Nevertheless, it is still the simplest form of the myoelectric control (i.e., only two channels, discrete control) that the subjects would have to master. With respect to aiming, there should be no significant difference between the ability of transradial amputees with the healthy elbow and upper arm, and the healthy population.

The requirement to aim the laser at the target object is a potentially counter intuitive step in the proposed approach. As shown in Fig. 3, the CVS was mounted above the hand, which is not an ideal position because the optical axes of the camera and the direction of the ultrasound burst are not aligned with the axis of the forearm. Therefore, the aiming with the laser had to be used to ensure that, in the initial phase of the movement, the user oriented the hand so that the target object was actually picked up by the sensors. However, the subjects in general had no difficulties in mastering this step. The aiming lasted from 2 to 3 seconds in average, even for the small objects. Furthermore, as will be explained later, the future goal is to miniaturize and integrate the CVS into the hand itself. In that case, the axes of the sensors would align with the axis of the forearm and the aiming could become automatic (subconscious), i.e., it could be an integral part of the approach phase during which the hand aligns with the target object in preparation for the grasp.

The outputs of the CVS are the estimated grasp type and aperture size appropriate for grasping the detected target object. Both outputs are essential for the successful grasping using an anthropomorphic artificial hand. If the grasp type is not adequate, it could be difficult to form a stable grip, as documented well in the studies on robotic grasping [36,39]. As demonstrated for the human [51] and robotic grasp planning [25,52], assuming a proper aperture plays a key role, i.e., forming an aperture with the size that is adapted to that of the object allows for a more accurate reaching and positioning of the hand and therefore leads to a better preparation for the following enclosing phase. This reasonably increases the chances of forming a stable grip.

The CVS was capable of generating nine different commands (i.e., combinations of grasp types and sizes) with a success rate of 84%. If the number of possible commands from the CVS was reduced, the success rate increased (up to 93%); thus, this control principle allows selection of the suitable trade-off between desired sophistication and robustness. In general, it is hard to define precisely and objectively what would be the acceptable performance for the eventual practical application (e.g., see the discussion of the "hot coffee problem" in [21]). Nevertheless, to reach a higher level of robustness, the current objective for the CVS classification is to improve the performance even further so that the error rates are reduced to below 5%. This can be done by improving the image processing and/or distance estimation as described later in the text.

The cognitive vision algorithm tested in this study was operating on the PC in the LabView environment. The processing of an image and the size estimation lasted an average of 0.3 seconds. As explained before, ten of these snapshots were taken and processed before the command could be sent to the hand. Overall, the period between the moment when the user issued a command (i.e., contracted his muscles) and the start of hand preshaping was too long: about 4 seconds (on average). Farrell and Weir [53] defined the notion of optimal controller-induced delay as the maximum amount of time that can be used by the controller for data collection and analysis without affecting prosthesis user performance. They also noted that there is no general agreement in the existing literature about the acceptable delays; the estimates in different studies range from as low as 50 to up to 400 milliseconds. One reason for this disagreement could be the fact that the acceptable delay likely depends on the specific control method used (e.g., proportional, discrete control) as well as on the mechanical characteristics of the prosthesis (e.g., speed of opening and closing). For the controller presented in this manuscript, the current goal is to decrease the delay so that it falls somewhere within the range of acceptable delays given in the literature (~few hundred milliseconds). This can be done by implementing the controller within an embedded platform and by optimizing the processing.

In the present study, unsuccessful grasps were caused by the three main reasons: 1) subjects made mistakes and failed trials while learning how to operate the system (in the initial trials, subjects wrongly positioned the hand around small objects; hence, the fingers missed the object while closing); 2) the CVS generated wrong commands due to wrong estimation of the object size; and 3) EMG triggering failed, especially in the initial trials while the subjects were still learning how to generate the commands. The wrong estimation of the object size was caused by the following: a) image segmentation and b) distance errors. We discuss these reasons below:

a) The segmentation is, in computer vision, the task of separating the object from the background; it is a crucial step in the algorithm since it allows the identification of the target object. During the experiments, the segmentation in some cases failed, "mistaking" a part of the background as a part of the object or vice versa. This led to a wrong estimation of the object axis lengths, that is, incorrect grasp type and/or size selection. Imperfect segmentation is a common problem in computer vision [54,55], and this limitation has already been identified in our earlier study [43]. As a result, in the present study, we improved the recognition by implementing an edge-based segmentation in the RGB colour space [56].

b) The wrong estimations were also caused by a false reading from the distance sensor: it sometimes registered a reflection from an object that was not the target (e.g., an edge of the table) or it missed the target object completely. The latter was the case when the target object was small. This error could be minimized by testing sensors equipped with different models of ultrasound transceivers [57] in order to find an optimal diameter of the cone of the emitted ultrasound burst (i.e., having a more or less focused beam).

Although the main goal of this study was to evaluate the performance of the control algorithm, we were also interested in assessing how easy it was to operate the system and if the subjects would improve their performance by learning how to use it. Results are encouraging, showing that the subjects were able to operate the system well just after a short period of practice (less than 15 minutes): all subjects failed less than five times (out of 18) in the first block of trials. Furthermore, in the second block, the subjects decreased the time needed to accomplish the task without actually sacrificing their performance. For example, the subjects learned that they could start reaching for the target object before the hand was fully preshaped into the selected grasp. Importantly, this is how the normal human grasp naturally develops; the transport and preshape components evolve in parallel.

It is important to emphasize that the goal of this work was to test the feasibility of the overall approach and not to test, fine tune and perfect all of its component parts. For example, the surface of the table and the wall behind the objects were in the plane colours during the experiments. Since the segmentation is based on edge detection, identifying an object in an image with a strongly textured background would be a much more challenging task. Due to similar reasons, the current algorithm would not perform well in cluttered environments, i.e., when there are many objects close and behind each other. Importantly, these issues are the focus of research in the computer vision community, and it is therefore to be expected that new solutions will soon emerge. Since the system proposed here is modular, the novel algorithms can be incorporated easily as soon as they appear.

In terms of aesthetics, again, the system shown in Fig. 3 should be regarded only as a first prototype. The CVS is presently mounted on the hand as a separate component; the sensor elements (e.g., lenses and image sensor, ultrasound transmitter and receiver) and their supporting electronics are housed in two metal boxes (see Fig. 3). However, the future goal is to integrate the CVS into a prosthetic hand. The sensor electronics will be merged with the electronics of the hand, and both will reside within the palm. The best positions for mounting the lenses and ultrasound transceivers (miniaturized versions) will be identified by testing. One possibility would be to place the components in the palm between the metacarpophalangeal joints. In the neutral hand posture, the fingers are somewhat flexed, and this would ensure a free line of sight. The cosmetic covering should have small openings for the sensors and the camera. Since the CVS implements a robust estimation rather than a fine and sensitive analysis, the new components will add a marginal load to the standard procedures for the hand maintenance (i.e., the lenses will have to be kept relatively clean). For one example of a camera that is actually a part of the robotic hand, see [58].

Future work will be focused mainly on developing a more robust computer vision part of the algorithm. For example, vector-based approaches for edge detection [56] or region growing methods [54] for object segmentation are possible directions for future research. However, these methods are significantly more demanding in terms of processing, and fast algorithms convenient for real-time implementation have yet to be developed. Regarding the hardware, the next step is to realize it as an embedded computer platform, such as the one presented in [59], and integrate it into a functional transradial prosthesis [3] in order to assess the usability of the approach in amputees.

## Conclusions

The original contribution of this research is a novel controller that uses vision and reasoning borrowed from biological control to implement high-level analysis (i.e., determining object properties) and autonomous decision-making (i.e., selecting appropriate grasp type and size). Importantly, the controller is designed to be convenient for real-time application: the image processing pipeline is minimal and the reasoning module comprises a set of simple IF-THEN rules. The automatic control eases the burden from the user; he/she is responsible for issuing just the basic commands of "open" and "close" via a simple two-channel EMG interface. As a result, the user can concentrate on what he/she does, not on how he/she should do it. The tests showed that the performance of the system was satisfactory and that the users could successfully operate the system with minimal prior training. Having an intelligent controller that operates autonomously while being integrated within the volitional control of the user, and thereby complementing the user in controlling the system, is essential for the implementation of complex control scenarios exploiting the full flexibility of the modern dexterous prosthetic hands.

## Competing interests

CC and MC hold Prensilia Srl, the company that manufactures robotic hands as the one used in this work, under the license to Scuola Superiore Sant'Anna.

## Authors' contributions

SD and CC contributed to all the stages of this research (i.e., planning, implementing, conducting experiments and writing). DBP conceived the concept of the novel control method, and participated in designing the control system, in planning the experiments, as well as in writing. MK contributed in the development of the image processing part of the control algorithm. MC and MCC developed the hand and took part in writing. All authors read and approved the final manuscript.

## Supplementary Material

Additional file 1 **IF-THEN rules**. The complete set of rules for selecting the grasp type and aperture size. Click here for file
