# Supplementary material for: Cognitive vision system for control of dexterous prosthetic hands: Experimental evaluation
Source: J Neuroeng Rehabil. 2010 Aug 23;7:42. doi: 10.1186/1743-0003-7-42 (PMC2940869; doi:10.1186/1743-0003-7-42)
Supplement: Additional file 1 — IF-THEN rules. The complete set of rules for selecting the grasp type and aperture size. [file 1743-0003-7-42-S1.PDF]

The set of IF–THEN rules for the selection of grasp type and aperture size

IF					THEN
$L \le T_{LARGE}$	AND	$S \le T_{VERYSMALL}$			2-digit
$L \le T_{LARGE}$	AND	$S > T_{VERYSMALL}$	AND	$S \le T_{SMALL}$	3-digit small
$L \le T_{LARGE}$	AND	$S > T_{SMALL}$			3-digit medium
$L > T_{LARGE}$	AND	$S \le T_{EXTRATHIN}$			lateral small
$L > T_{LARGE}$	AND	$S > T_{EXTRATHIN}$	AND	$S \le T_{VERYTHIN}$	lateral medium
$L > T_{LARGE}$	AND	$S > T_{VERYTHIN}$	AND	$S \le T_{THIN}$	lateral large
$L > T_{LARGE}$	AND	$S > T_{THIN}$	AND	$S \le T_{WIDE}$	palmar small
$L > T_{LARGE}$	AND	$S > T_{WIDE}$	AND	$S \le T_{VERYWIDE}$	palmar medium
$L > T_{LARGE}$	AND	$S > T_{VERYWIDE}$			palmar large

The numerical thresholds for the rules were adopted as follows:

T_{LARGE} = 90% PW; T_{WIDE} = 50% MPA; $T_{VERYWIDE}$ = 65% MPA;
T_{THIN} = 70% MLA; $T_{VERYTHIN}$ = 40% MLA; $T_{EXTRATHIN}$ = 30%MLA;
T_{SMALL} = 30% MPA; $T_{VERYSMALL}$ = 15% MPA;

The notations are: *L*, *S* – long and short object axes; *MPA* – maximal palmar aperture (measured as the distance from the tip of the thumb to the tip of the index finger; the hand was preshaped for the palmar grasp with the fingers maximally extended); *MLA* – maximal lateral aperture (measured as the distance from the tip of the thumb to the radial side of the index finger; the hand was preshaped for the lateral grasp with the thumb maximally extended); PW – palm width (measured as the distance from the radial aspect of the metacarpophalangeal joint of the index finger to the ulnar aspect of the metacarpophalangeal joint of the little finger).
